# Genome sequencing and identification of cellulase genes in *Bacillus paralicheniformis* strains from the Red Sea

**DOI:** 10.1186/s12866-021-02316-w

**Published:** 2021-09-22

**Authors:** Siham Fatani, Yoshimoto Saito, Mohammed Alarawi, Takashi Gojobori, Katsuhiko Mineta

**Affiliations:** 1 Computational Bioscience Research Center (CBRC), King Abdullah University of Science and Technology (KAUST), Thuwal, Saudi Arabia; 2 Marine Open Innovation Institute (MaOI), Shizuoka, Japan

**Keywords:** Cellulase, The Red Sea, Bioprospecting, Cellulolysis, Operon, Whole genome sequencing, Gene expression analysis

## Abstract

**Background:**

Cellulolytic microorganisms are considered a key player in the degradation of plant biomass in various environments. These microorganisms can be isolated from various environments, such as soils, the insect gut, the mammalian rumen and oceans. The Red Sea exhibits a unique environment in terms of presenting a high seawater temperature, high salinity, low nutrient levels and high biodiversity. However, there is little information regarding cellulase genes in the Red Sea environment. This study aimed to examine whether the Red Sea can be a resource for the bioprospecting of microbial cellulases by isolating cellulase-producing microorganisms from the Red Sea environment and characterizing cellulase genes.

**Results:**

Three bacterial strains were successfully isolated from the plankton fraction and the surface of seagrass. The isolated strains were identified as *Bacillus paralicheniformis* and showed strong cellulase activity. These results suggested that these three isolates secreted active cellulases. By whole genome sequencing, we found 10 cellulase genes from the three isolates. We compared the expression of these cellulase genes under cellulase-inducing and non-inducing conditions and found that most of the cellulase genes were generally upregulated during cellulolysis in the isolates. Our operon structure analysis also showed that cellulase genes form operons with genes involved in various kinds of cellular reactions, such as protein metabolism, which suggests the existence of crosstalk between cellulolysis and other metabolic pathways in the bacterial isolates. These results suggest that multiple cellulases are playing important roles in cellulolysis.

**Conclusions:**

Our study reports the isolation and characterization of cellulase-producing bacteria from the Red Sea. Our whole-genome sequencing classified our three isolates as *Bacillus paralicheniformis*, and we revealed the presence of ten cellulase orthologues in each of three isolates’ genomes. Our comparative expression analysis also identified that most of the cellulase genes were upregulated under the inducing conditions in general. Although cellulases have been roughly classified into three enzyme groups of beta-glucosidase, endo-β-1,4-glucanase and exoglucanase, these findings suggest the importance to consider microbial cellulolysis as a more complex reaction with various kinds of cellulase enzymes.

**Supplementary Information:**

The online version contains supplementary material available at 10.1186/s12866-021-02316-w.

## Background

Cellulose, which is the major component of plant biomass, is the most abundant organic compound on Earth and a sustainable source of energy [1]. It is composed of a linear homologous polymer chain consisting of D-glucose residues, containing up to 10,000 glucose residues linked by β-1,4-glycosidic bonds [2, 3]. The efficient conversion of cellulose into its glucose monomers by microbes as a source of high-energy molecules helps to meet future energy needs and serves as an alternative source of renewable energy [4]. The biodegradation of β-1,4-glycosidic bonds in cellulose biomass is carried out by free cellulases or a multienzyme complex referred to as the cellulosome, which can catalyze the hydrolysis of cellulose into sugars. These enzymes are produced by various microorganisms, such as bacteria and fungi [5].

Several cellulase-producing microorganisms with high cellulolytic activity have been isolated from the fungal genera *Aspergillus* and *Trichoderma* [6]. Cellulase activity has also been observed in bacterial genera including *Alteromonas, Acetivibrio, Bacillus, Bacteroides, Cellulomonas, Clostridium*, and *Ruminococcus* [7]. *Bacillus* species have been employed for production of cellulase [8, 9]. As represented by *B. subtilis*, a lot of studies regarding cellulase gene sequences, enzymatic activities, optimal condition for cellulolysis were published from *Bacillus* species. Cellulases from *Bacillus* are still now reported frequently [10, 11].

Cellulases are a group of three types of enzymes with different activities. The first type is endo-β-1,4-glucanase (EC 3.2.1.4), which can perform cleavage on internal bonds in the cellulose fibers. The second is exoglucanase (EC 3.2.1.91), which binds at the reducing or non-reducing ends of cellulose fibers and cleaves them to produce short disaccharides [12]. The third is β-glucosidase (EC 3.2.1.21), which hydrolyzes cellobioses to produce glucose molecules [8]. Recent studies have classified the cellulases of all three types as glycoside-hydrolases (GHs). The glycoside-hydrolase is a group of hydrolases composed of a great number of enzymes. The Carbohydrate-Active Enzymes CAZy database [13] provides that the cellulases reported thus far are classified into of 16 widely ranging GH orthologous groups.

In contrast to the terrestrial environment, a small number of studies have been conducted to investigate cellulase-producing microorganisms in marine environments. However, the isolation and characterization of cellulases from marine bacteria are now more frequently reported [8]. Studies regarding marine cellulases have revealed characteristic of these enzymes with possible application of the enzyme and producing strains.

The Red Sea has unique marine environmental features compared to the other oceans. The Red Sea has been described as an oligotrophic environment consisting of one of the warmest and saltiest water bodies in the world, with year-round high UV radiation [14]. These characteristics are thought to have given rise to and modulated the evolution and diversity of microbial forms in the Red Sea. To our knowledge, cellulase-producing microorganisms from the Red Sea environment remain unstudied. In particular, the diversity, abundance, and characteristics of cellulase genes or cellulolytic microorganisms are still far from fully explored.

In this study, cellulase-producing bacteria were isolated from the Red Sea environment. We also identified cellulase genes and revealed that they were expressed during cellulolysis and provided important information to understand the mechanism of microbial cellulolysis in detail.

## Results

### Isolation and Screening of Cellulase-Producing Microorganisms

Surface seawater samples were collected from a coastal region of the Red Sea at Thuwal in Saudi Arabia. The samples were diluted and spread on Nutrient Media (NM) plates and incubated at 30˚C following a previously reported protocol [15]. Four hundred fifty-six colonies were isolated on NM plates and subsequently streaked on media containing Carboxymethyl cellulose (CMC) as the sole carbon source for the screening of cellulase-producing microorganisms. No isolates showed cellulase activity on the CMC medium plates (Table 1).

**Table 1:** Screening of cellulase-producing bacteria from seawater, seagrass and plankton fraction samples

Sample	Seawater	Seagrass		Plankton
No. of picked colonies from NM plate	456	126	60	
No. of clones showing cellulase activity on CMC plate	0	2	1	

*NM* nutrient media; *CMC* carboxymethyl cellulose

**Fig. 1:**
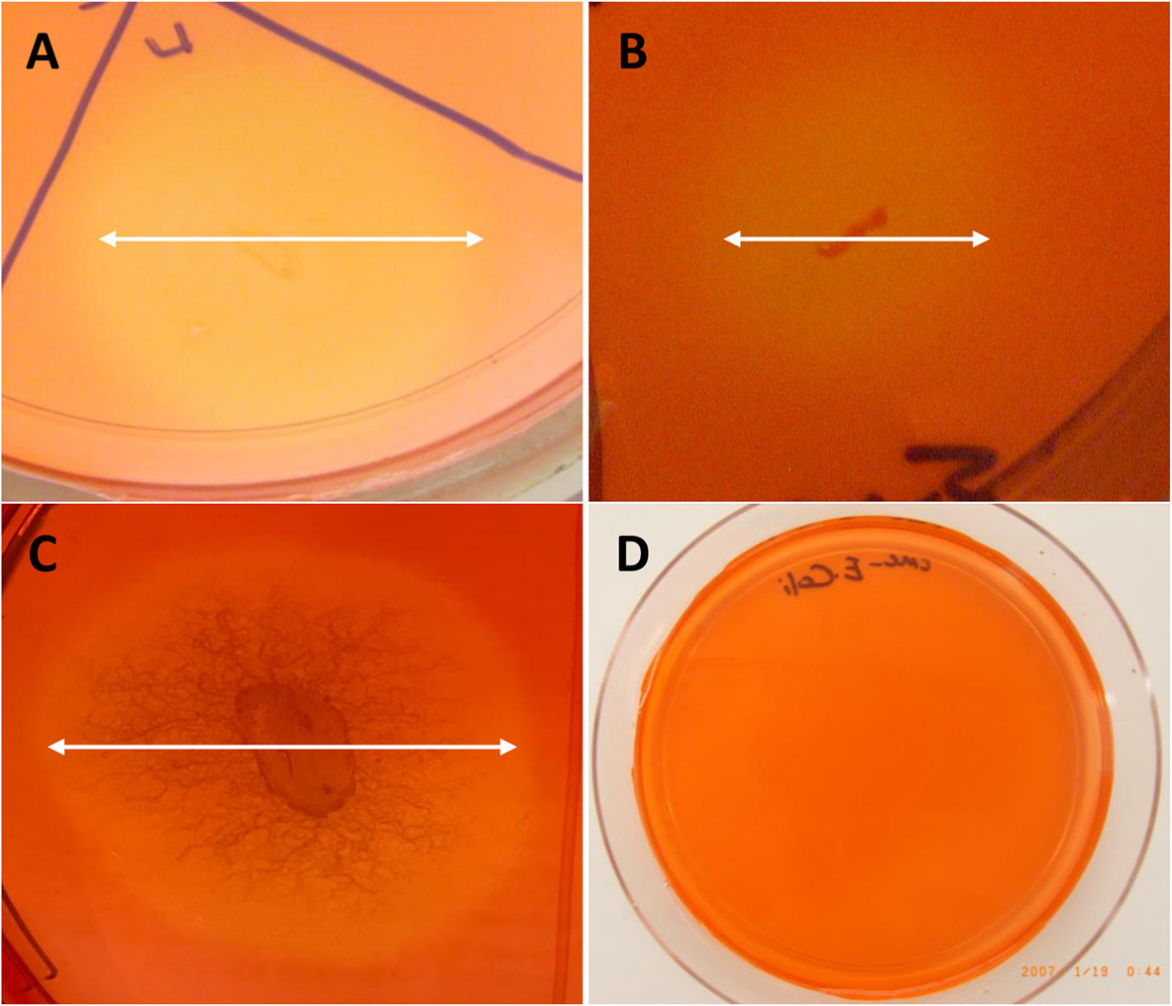
Screening of cellulase-producing microorganisms by using Congo red on CMC agar plates. (**A**) Bacterial isolate from the plankton sample (PB1), (**B** and **C**) bacterial isolates from the seagrass samples (SB2 and SB3) and (**D**) *Escherichia coli* as a negative control

We then used the plankton fractions obtained from surface seawater as the source for the isolation of cellulase-producing microorganisms. Sixty strains were isolated on NM plates, and one isolate (PB1) formed a zone of clearance around the colonies on CMC medium plates after staining with Congo Red (Fig. 1). We also tried to isolate cellulase-producing bacteria from the surface of green seagrass. One hundred and twenty-six strains were obtained on NM plates, and two strains (SB2, SB3) showed a clear halo zone on CMC medium plates after staining (Table 1). We then characterized these three strains as cellulase-producing isolates from the Red Sea.

### Cellulase enzyme assay

**Fig. 2:**
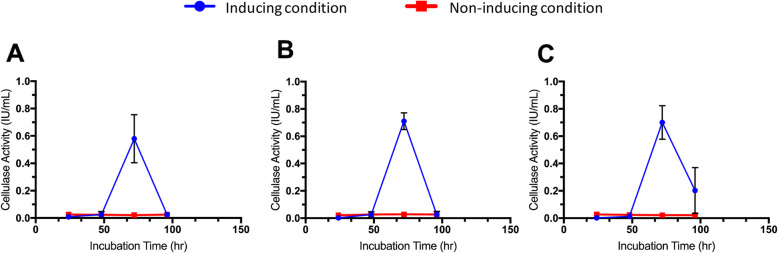
Measurement of the cellulase activity of the three bacterial strains under inducing and non-inducing conditions via filter paper assays. (**A**) PB1, (**B**) SB2 and (**C**) SB3

The measurement of cellulase activities for the three strains was in broth media that containing a strip of cellulose filter paper as the sole carbon source. The complete degradation of the filter paper was observed in all the PB1, SB2 and SB3 cultures whereas the control (i.e., the same culture conditions without the bacterial inoculate) showed very slight degradation of the filter paper (Fig. S1). The measurement of the amount of reducing sugar formed in broth media after four days of cultivation revealed that all three strains showed maximum cellulase activity after 72 h of incubation (Fig. 2). The maximum activities were 0.75, 0.59 and 0.70 filter paper unit (FPU)/ml in the PB1, SB2 and SB3 isolates, respectively.

### Growth test under high salinity conditions

We investigated whether our three isolates from the Red Sea have abilities to do cellulolysis under high salinity condition. First, we tested the growth of the isolates in NM broth under the salinity conditions ranging from 0.2 % (34mM) to 10 % (1.7 M) at 24 h after inoculation. Although the growth of the three strains gradually retarded along with the concentration of NaCl, the results clearly showed that OD_600_ of the isolates were 0.5–0.9 at the concentration of 8 % (1.4 M) and 0.12–0.36 at 10 % (1.7 M) NaCl (Fig. S2a). Next, we tested the growth of the isolates on CMC broth where they are able to use CMC only as a carbon source under various salinity stress conditions at 48 h after inoculation. Compared with NM broth, the isolates showed lower OD_600_ values in CMC broth at all the NaCl concentrations. However, the isolates still showed the growth (around 0.1 of OD_600_) in CMC broth under 8 % salinity (Fig. S2b, Fig. S2c).

### Whole-Genome Sequencing of Bacterial Isolates

The genome sequences of the three strains were determined using the PacBio RSII platform. The average of sequencing coverage was 186x. The obtained sequences were *de novo* assembled as a single circular chromosome in each strain, and no plasmids were detected in their genomes. The total genome sizes of PB1, SB2 and SB3 were 4,318,221, 4,318,038, and 4,317,481 bp, respectively (Table S1). We then conducted gene prediction, resulting in the prediction of 4362, 4441 and 4675 genes in the genomes of the three isolates, respectively.

### Multilocus Sequence Typing (MLST) Analysis

**Fig. 3:**
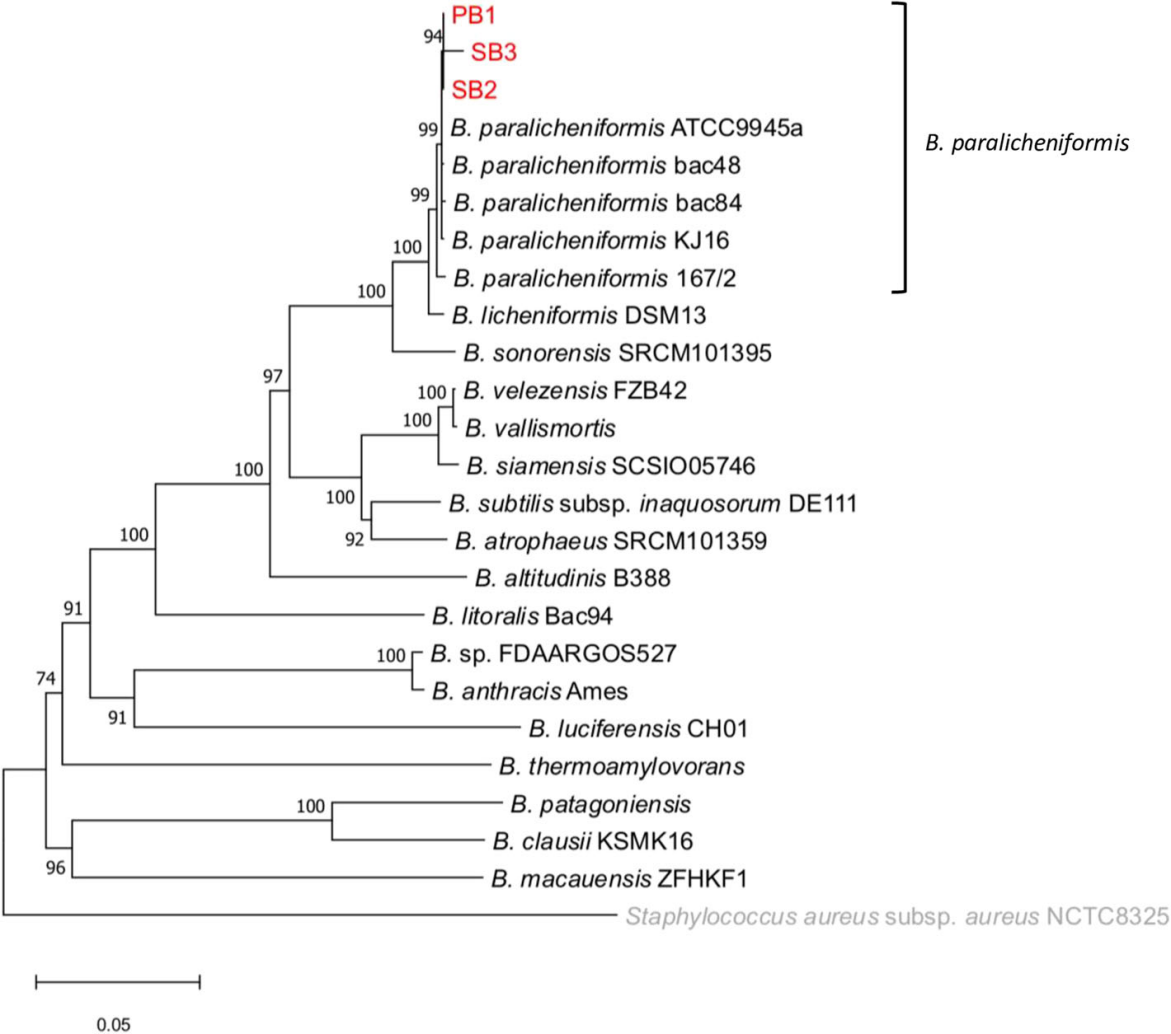
Neighbor-joining phylogenetic tree based on MLST housekeeping genes in PB1, SB2 and SB3 and other *Bacillus* strains. The value at each node represents the bootstrap value (1,000 replicates). The units of the bar indicating the evolutionary distance are the number of nucleotide substitutions per site

To determine the phylogenetic positions of the three strains within genus *Bacillus*, we conducted MLST by using thirteen housekeeping genes amino acid sequences (adk, ccpA, recF, rpoB, spo0A, sucC, glpF, ilvD, pta, purH, pycA, rpoD, tpiA and gmk). The three bacterial strains were all included in the cluster of *Bacillus paralicheniformis* strains with a 100 % bootstrap value. Furthermore, the three isolates formed a cluster with 94 % bootstrap support (Fig. 3).

### Identification of Cellulase Genes in the Isolates’ genomes

We examined GH functional domains in the amino acid sequences of predicted genes and found 10, 10 and 11 cellulase genes from PB1, SB2 and SB3 strain genomes, respectively. Based on the similarity, these genes were classified into 10 orthologues and designated as Cellulase 1 to 10 (Cel-1 to Cel-10) respectively (Table 2). Amino acid sequences of each cellulase orthologue were 100 % identical among three isolates. The isolates possess each orthologue as a single copy in general. Only SB3 has exceptionally two Cel-10 orthologues, GENE_1822 and GENE_1823. It is noteworthy that the amino acid sequence of GENE_1822 shows identity to the former part of Cel-10 orthologues in PB1 and SB2, while GENE_1823 is identical to the latter part of Ce1-10 (Fig. S3). Considering homologies to GH domain, orthologues of Cel-1, Cel-2, Cel-3, Cel-4 and Cel-5 were annotated as members of the GH1 family while Cel-6, Cel-7, Cel-8, Cel-9 and Cel-10 were predicted as GH3, GH5, GH9, GH26 and GH48 cellulases, respectively (Table 2).

**Table 2:** Cellulase genes in the three isolates

Cellulase orthologues	Genes in PB1	Genes in SB2	Genes in SB3	GH family	Cellulase activity
**Cel-1**	GENE_769	GENE_2609	GENE_4396	GH1	β-glucosidase/exoglucanase
**Cel-2**	GENE_743	GENE_2582	GENE_4369	GH1	
**Cel-3**	GENE_1298	GENE_3145	GENE_288	GH1	
**Cel-4**	GENE_718	GENE_2557	GENE_4343	GH1	
**Cel-5**	GENE_3516	GENE_958	GENE_2665	GH1	
**Cel-6**	GENE_1100	GENE_2942	GENE_74	GH3	β-glucosidase
**Cel-7**	GENE_3008	GENE_443	GENE_2102	GH5	Endo-β-1,4-glucanase
**Cel-8**	GENE_2740	GENE_173	GENE_1821	GH9	Endo-β-1,4-glucanase/ β-glucosidase/ exoglucanase
**Cel-9**	GENE_1657	GENE_3511	GENE_679	GH26	Endo-β-1,4-glucanase
**Cel-10**	GENE_2741	GENE_174	GENE_1822	GH48	Endo-β-1,4-glucanase/ exoglucanase

### Identification of Operon Structure

**Fig. 4:**
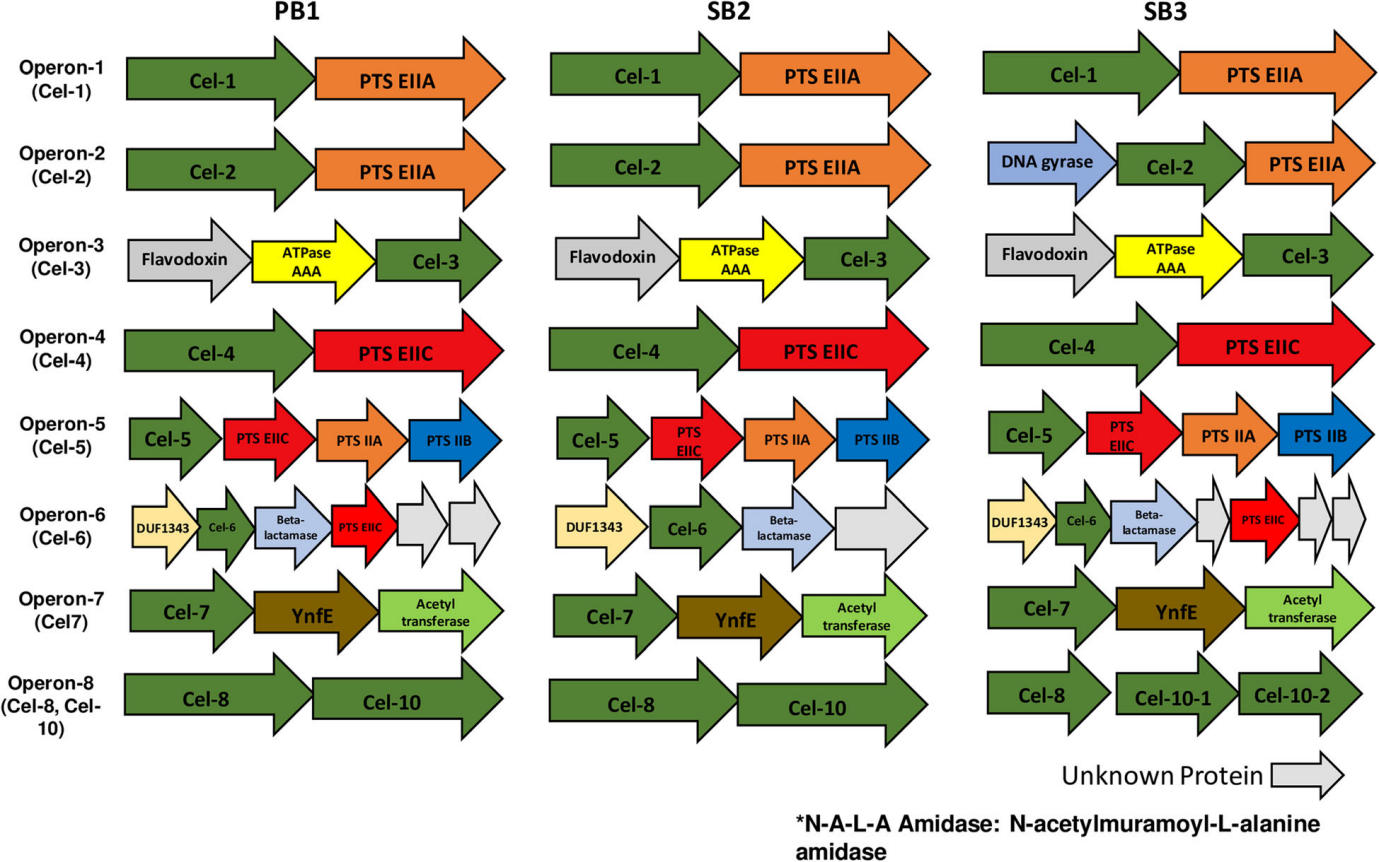
Operon structure of cellulase genes in PB1, SB2 and SB3

To understand what kind of genes were co-regulated with these cellulase genes, we surveyed structures of operons containing cellulase genes we found. As shown in Fig. 4, nine of the ten cellulase orthologues formed operons. We designated them as Operon-1 to Operon-8. Here, Cel-8 and Cel-10 appeared in the same operons, Operon-8. The structures of these eight operons were generally conserved in the three isolates.

Cel-1, Cel-2 Cel-4 and Cel-5 orthologues, all of which belonged to GH1 cellulase (β-glucosidase/exoglucanase), formed operons with the phosphoenolpyruvate-dependent sugar phosphotransferase system (PTS) components. However, inferred functions of those PTSs were different among each operon. PTS component in the Operon-1 showed 77 % identity in amino acid sequence with the PTS component of the sucrose transporting in *B. subtilis*, BglP while the sequence of the PTS component in Operon-2 was similar to the *B. subtilis* glucose-transporting PTS component, PtsG with 60.8 % identity [16].

Operon-5 also included three PTS components with the cellulase Cel-5. These three PTS proteins showed 76 %, 70 and 82 % sequence identities with cellobiose importing PTS components in *B. subtilis*, LicC (CelB), LicB (CelA) and LicA (CelC), respectively [17]. Cel-4 also formed with a PTS component in Operon-4, however, this protein did not show high homology with any known PTS components in *B. subtilis*. It only showed 32 % amino acid sequence identities with LicC of *B. subtilis*.

On the other hand, Operon-3, which also included the GH1-type β-glucosidase Cel-3, did not contain PTS components. Genes encoding flavodoxin and AAA-type ATPase were included in this operon, instead [15].

Cel-6, another type of β-glucosidase (GH3), formed operons with two genes in Operon-6. The one encoded a protein with the conserved unknown functional domain DUF1343 (Pfam) and the other is predicted to be a N-acetylmuramyl-L-alanine amidase gene [18].

Cel-7, which was classified as an endo-β-1,4-glucanase (GH5), formed Operon-7 with a function-unknown protein YnfE and acetyltransferase genes. The two cellulase, Cel-8 and Cel-10, which were classified as endo-β-1,4-glucanase of GH9 and GH48 respectively, formed Operon-8 together in all the three isolates. On the other hand, only Cel-9, which encoded GH26-type endo-β-1,4-glucanase, was not included in any operons in all three isolates. It is also revealed that expression of genes included in the same operon were generally co-regulated in these three isolates (Table S2).

### Comparative Expression Analysis of Cellulase Genes in the Isolates

To reveal whether the identified 10 cellulase genes are actually functional during cellulolysis, we conducted comparative expression analysis. We investigated expression of each gene under two different culture conditions: cellulase-inducing and non-inducing conditions. Enzymatic activity of cellulase was observed under cellulase-inducing conditions, i.e., after 72 h of incubation when it reached the maximum cellulase activity, while no cellulase activity was detected under the non-inducing conditions in all strains (Fig. 2). Because of the low number of mapped reads at PB1 (Table S3), we only focused on SB2 and SB3. The results revealed that 9 of 10 cellulase genes were upregulated in both SB2 and SB3 under the cellulase-inducing conditions (Table 3). In addition, each strain exhibited particular genes that were particularly highly upregulated under the cellulase-inducing conditions (i.e., showing ten-fold higher expression than under non-inducing conditions) as below.

**Table 3:** Comparative expression analysis of cellulase genes in three isolates

		SB2				SB3			
**Orthologue**	**GH**	**Gene**	**Inducing**	**Non** **inducing**	**Ratio**	**Gene**	**Inducing**	**Non** **inducing**	**Ratio**
**Cel-1**	GH1	GENE_2609	188.18	81.24	2.32	GENE_4396	3.23	0.18	18.22
**Cel-2**	GH1	GENE_2582	66.47	37.67	1.76	GENE_4369	347.44	17.03	20.40
**Cel-3**	GH1	GENE_3145	15.46	7.49	2.07	GENE_288	157.71	7.62	20.70
**Cel-4**	GH1	GENE_2557	7.90	2.82	2.80	GENE_4343	19.90	0.00	CMC
**Cel-5**	GH1	GENE_958	949.03	2.29	414.79	GENE_2665	21.05	6.57	3.21
**Cel-6**	GH3	GENE_2942	30.19	0.78	38.78	GENE_74	261.19	0.91	288.46
**Cel-7**	GH5	GENE_443	48.48	261.73	0.19	GENE_2102	4.23	2.26	1.87
**Cel-8**	GH9	GENE_173	1.23	0.35	3.48	GENE_1821	43.32	2.08	20.87
**Cel-9**	GH26	GENE_3511	21.97	6.73	3.26	GENE_679	13.55	7.48	1.81
**Cel-10**	GH48	GENE_174	4.51	0.66	6.87	GENE_1822	16.64	4.61	3.61

Values in inducing and non-inducing columns represent RPKM in each condition. Ratio columns represents ratio of RPKM values in these two conditions. ”CMC” at the ratio column means the expression specific to the non-inducing condition

In the SB2 strain, the expression of Cel-5 (GH1) and Cel-6 (GH3) was highly upregulated. In the SB3 strain, four genes belonging to the GH1 family, Cel-1, Cel-2, Cel-3 and Cel-4, were highly expressed or expressed specifically in the inducing condition. Cel-6 (GH3) and Cel-8 (GH9) were also highly expressed in this isolate. In addition, one of the two Cel-10 (GH48) orthologues in SB3, GENE_1822 showed the expression pattern specific to the inducing condition.

## Discussion

### Isolation of Cellulase-Producing Microorganisms

Cellulase-producing microorganisms are usually isolated from terrestrial environments [19], whereas there are only a few reports regarding the isolation of these microorganisms from a seawater environment [20]. Harshvardhan et al. (2013) reported the isolation of the cellulolytic marine species *Bacillus* sp. H1666 from seawater samples along the western Indian coast [21]. Additionally, Samira et al. (2011) reported the isolation of *Stenotrophomonas maltophilia* from surface seawater in the Persian Gulf [22]. In our study, we could not isolate any cellulase-producing microorganisms from the surface seawater of the Red Sea. On the other hand, we report the isolation of two bacterial strains from the surface of seagrass.

This result indicates that cellulase-producing microorganisms are usually present in association with cellulose-containing substrates such as seagrass [23], which might also explain the difficulty in isolating cellulase-producing microorganisms from shallow seawater. Previous studies supporting our results such as that of Trivedi et al. (2011) have also reported the isolation of *Bacillus aquimaris*, *B. flexus* NT and *Pseudoalteromonas* CSMCRI-5 strains with cellulolytic potential from green seagrass [4].

In addition, we isolated one cellulase-producing strain from the plankton fraction (i.e., samples collected from the surface seawater in which phytoplankton cells were condensed), suggesting that although the number of phytoplankton cells was very low, cellulase-producing bacteria probably live on the surface of phytoplankton cells in the Red Sea surface waters. To date, there have been no reports of the isolation of cellulase-producing microorganisms from plankton fractions; therefore, our PB1 strain is the first isolate of a cellulase-producing microorganism associated with marine plankton.

Our growth test revealed that the three isolates are able to grow well at the concentration of 8 % NaCl and still slightly proliferated at 10 % NaCl in NM broth. The tolerances of the isolates against salinity are not at the same level with those of so-called halotolerant strains like *B. subtilis* strain FP-133 since they can grow at the concentration higher than 12.5 % NaCl [24]. However, Schroeter et al. reported that *B. lincheniformis* DSM 13 did not grow at the concentration of more than 8 % (1.4 M) NaCl [25]. Although the culturing conditions are different between Schroeter et al.’s study and ours, the isolates might be tolerant against salinity moderately. Our results also revealed that the isolates showed the growth in CMC broth at the concentration of 8 % NaCl, which allows us to expect that the isolates can degrade the cellulose under high salinity conditions. These might be a feature of cellulase producing bacteria in the Red Sea.

### Taxonomic Prediction and cellulase activity measurement

*B. paralicheniformis* was recently described as a new species of genus *Bacillus* and isolated from various environments, including marine, freshwater, and food-related niches. In a previous study, the isolation of the bacteria *B. paralicheniformis* bac48 and bac84 from the Red Sea environment was reported by Othoum et al. (2018) [26]. Dass et al. (2018) isolated *B. paralicheniformis* F47 from a salty lake in Algeria [27]. Here, our MLST analysis showed that our three strains were closely related to *B. paralicheniformis* species. Although cellulases remain unexplored in *B. paralicheniformis* species, this species including our isolates might potentially be adaptive to high salinity environments under the hot climates such as the Red Sea. Our strains showed cellulase activities of 0.75 FPU/ml, 0.70 FPU/ml and 0.59 FPU/ml, respectively. Although no common criteria (unit) for evaluating cellulase activity have been developed, Samira et al. (2011) measured the cellulase activities using the same method as ours except the type of the buffer (see Materials and Methods). They measured the activities of three marine bacterial isolates obtained from the Arabian/Persian Gulf and reported activities of 0.079, 0.074, and 0.072 FPU/ml for their strains. Our isolates showed more than ten times higher cellulase activity than their strains [18].

### Identification of Cellulase Genes in the Isolates’ genomes

We also identified cellulase genes expected to be responsible for cellulolysis in each strain. Recent genome sequencing projects in cellulase-producing microorganisms have revealed the presence of several cellulase genes in their genomes [28]. The genomic analysis of our strains revealed that these three isolates possess the gene set of ten cellulase orthologues in general, and the amino acid sequences of each cellulase orthologue were identical among these three isolates. Regarding Cel-10, PB1 and SB2 have only one orthologue while SB3 has two Cel-10 orthologues. Either of Cel-10 sequences in SB3 was short and almost identical to the former or the latter part of the other isolates’ Cel-10 sequences. Therefore, we should take into account the possibility of misannotation and/or sequencing errors in the genomic region including two Cel-10 orthologues in SB2, although this study conducted genome sequencing with PacBio RS II platform at 186 x coverage, allowing us to expect the high accuracy of resultant genome sequences. As far as we surveyed, this is the first report on the active cellulases in *B. paralicheniformis.*

### Operon Structure Identification and Comparative Expression Analysis of Cellulase Genes in the Isolates

To determine cellulase genes expressed during cellulolysis, we conducted comparative expression analysis between two different conditions: cellulase-inducing and non-inducing conditions. Our comparative expression analysis showed that 9 of 10 cellulase genes were upregulated in SB2 and SB3 under the inducing condition. In this analysis, we omitted PB1 as the number of RNA-seq short reads mapped on open reading frame (ORF) regions in PB1 under the inducing condition was much smaller than those in the other strains (Table S3).

This study also revealed that particular types of cellulase genes were highly upregulated during cellulolysis. Most of these genes were predicted to encode β-glucosidases/exoglucanase classified as GH1 family. Our operon structure analysis identified that most of the GH1 cellulase genes formed operons with PTS system components involved in the import of various kind of sugars such as, sucrose, glucose and cellobiose [29–31]. The GH1 cellulase Cel-3 gene were also found to be included in the operon with flavodoxin and ATPases AAA protein, which is expected to be involved in diverse cellular activities [32]. Cel-6 gene, which encoded a β-glucosidase of GH3 family, were commonly included in the operon with a N-acetylmuramyl-L-alanine amidase gene showing 63.2 % identity with AmiE of *B. subtilis.* AmiE is revealed to be involved in the pathway of peptidoglycan recycling and in cell wall biogenesis [33]. These results suggest that these highly upregulated cellulase genes were co-regulated together with various genes present in the same operon, which enhanced a wide range of cellular reactions during cellulolysis. It is also noteworthy that the gene sets of highly upregulated GH1 β-glucosidases genes were slightly different from each other among the three orthologues, indicating that the strain-specific intracellular regulation might occur during the cellulolysis in each isolated.

On the other hand, no significant upregulation of endo- β-1,4-glucanases were observed during the cellulolysis in the expression analysis, although we confirmed the clear cellulolytic activity from the samples we used for the RNA-sEq. It may be because the expression peak of endo- β-1,4-glucanase genes had come a little bit earlier than the peak of enzymatic activity. The cellulase activity was measured by the amount of reducing sugars generated as the final product of the filter paper degradation. β-glucosidases catalyze the division of disaccharides to monosaccharides. In particular, GH3 β-glucosidases were predicted to be a secreted protein while the other β-glucosidases (i.e., GH1 proteins) were predicted to be localized inside the cell (Table S4). GH3 β-glucosidases were highly upregulated in all three isolates under the inducing condition, and the gene products were probably catalyzing the degradation of disaccharides outside the cell. High activity of GH3 β-glucosidases is consistent with the observation of the highest enzymatic activities at the same timing. More detailed time course settings may be required to characterize the expression pattern of endo-β-1,4-glucanases during microbial cellulolysis.

## Conclusions

Three cellulase-producing bacteria were obtained from the plankton fraction and seagrass surface in the Red Sea environment. We identified ten cellulase genes in their genomes and revealed that those genes are expressed during cellulolysis. The Red Sea exhibits high salinity (36–40 p.s.u.) and high surface temperatures (24 °C in spring and up to 35 °C in summer) [14]. The isolates obtained in this study are expected to produce cellulases that may be stable under such harsh conditions. Further analysis will provide valuable information on microbial cellulases in the Red Sea, which will contribute to industrial applications such as the development of plant biomass biorefineries.

## Methods

### Collection of Samples from the Red Sea

Marine samples were collected from a coastal region of the Red Sea at Thuwal in Saudi Arabia on August 26 and September 30, 2015 for the isolation of cellulase-producing microorganisms. A seawater sample was obtained from the seawater surface at the site at 22°17.444’N, 39°03.183’E using a Niskin bottle. A seagrass (sargassum weed) sample was obtained from the KAUST coastal marina (22°18’16.7"N 39°06’12.1"E), and a plankton sample was collected from the sea surface by drawing a net with a mesh size of 0.63 μm at 1 knot for 10 min. All samples were placed in sterile tubes and stored at 4˚C until use. The sample collection was followed by the institutional field research policy and procedure.

### Isolation and Screening of Cellulase-Producing Microorganisms

The seawater and plankton samples were vortexed for 15 min in sterilized 50 ml tubes and then allowed to settle for 5 min. Ten-fold serial dilutions of each sample were prepared in sterilized distilled water, and 0.1 ml diluted samples were spread on the surface of NM plates containing 0.3 % beef extract, 0.5 % peptone, 0.5 % NaCl, and 1.7 % agar [pH 7.0]. The plates were incubated at 30 °C for 48 h. One gram of seagrass was also measured and added to 10 ml of sterilized water. One gram of glass beads (425–600 μm) was placed in 1 M HCl for 1 h and then rinsed with distilled water. The resultant acid-washed glass beads were added to the seagrass tubes, which were then vortexed for 10 min. The tubes were left for 5 min to allow the solids to settle, and the supernatant was collected and diluted to use it as an inoculation source and plated on NM plates [15].

To screen for cellulase-producing microorganisms, single colonies from NM were transferred to CMC agar composed of 0.2 % NaNO_3_, 0.1 % K_2_HPO_4_, 0.05 % MgSO_4_, 0.05 % KCl, 0.2 % CMC sodium salt, 0.02 % peptone, and 1.7 % agar. Following it, the plates were incubated at 30˚C for 48 h. Zones of hydrolysis were visualized by flooding the plates with 0.1 % Congo red for 20 min and then washing the plates with 1 M NaCl for 20 min [15].

### Preparation of Extracellular Cellulase Enzymes

The obtained cellulase-producing strains were precultured in 100 ml of nutrient broth and incubated at 30˚C for 48 h at 200 rpm. Aliquot of 2 ml was used as the inoculum for enzyme production, and the broth culture system was composed of 0.2 % NaNO_3_, 0.1 % K_2_HPO_4_, 0.05 % MgSO_4_, 0.05 % KCl, 0.02 % peptone and a Whatman No. 1 Filter paper (1 × 6 cm strip, 0.05 g per 20 ml) [34]. The broth cultures were incubated for four days at 30˚C with shaking at 200 rpm. Cell growth was monitored every 24 h by determining the optical density at 600 nm, and cellulase activity was measured every 24 h during incubation. The cultures were centrifuged at 8,000 rpm for 10 min, and the supernatant was used as a source of crude enzyme for the determination of enzyme activity.

### Growth test under increasing salinity condition

The growth of the three bacterial isolates under high salt condition (i.e., 0.2 %, 2 %, 4 % 6 % 8 and 10 % (w/v) of NaCl) was studied in Nutrient broth media (NM) and compared with CMC broth media (i.e., where the cellulose is a sole carbon source).

Six Nutrient broth media were prepared which composed of (0.3 % beef extract, 0.5 % peptone, and NaCl (i.e., 0.2 %, 2 %, 4 % 6 % 8 and 10 % (w/v)). Likewise, for the CMC broth media, six media were prepared that contain (0.2 % NaNO_3_, 0.1 % K_2_HPO_4_, 0.05 % MgSO_4_, 0.05 % KCl, 0.02 % peptone, CMC 0.2 % and six different concentrations of NaCl i.e., (0.2 %, 2 %, 4 % 6 % 8 and 10 % (w/v). The broth media were incubated at 30 °C with shaking at 200 rpm. Cell growth was monitored every 24 h up to 5 days by determining the optical density at 600 nm.

### Measurement of Cellulase Activity

The filter paper assay of Hankin and Anagnostakis was used to measure total cellulase activity in the culture [5]. Total cellulase activity was determined by measuring the amount of reducing sugar formed by the degradation of filter paper strips. Then, 0.5 mL of the supernatant of the culture was incubated in 1.0 mL of 0.05 M sodium citrate buffer (pH 4.8) with a Whatman No. 1 filter paper strip, 1.0 × 6.0 cm (= 50 mg). After incubation for one hour at 50˚C, the reaction was stopped by adding 3 ml of dinitrosalicylic acid to the reaction mixture [35]. The amount of reducing sugars released was estimated spectrophotometrically at 540 nm using glucose as a standard. The enzymatic activity of total cellulases was defined in FPU/ml. One unit of cellulase activity is defined as the amount of enzyme releasing 1 µmol of reducing sugars (measured as glucose) from filter paper per mL per min [34].

### Whole-Genome Sequencing

DNA samples for the whole-genome sequencing were prepared by culturing the isolates in nutrient broth overnight at 30˚C with shaking at 150 rpm. DNAs were extracted from the isolates using the Qiagen DNeasy Blood & Tissue Kit following the manufacturer’s instructions [36]. The obtained DNA was quantified with a Qubit dsDNA BR assay kit (Thermo Fisher Scientific). Electrophoresis was also performed in a 1 % agarose gel to confirm that the length of the DNAs was longer than 40 Kb. Fifty micrograms of DNA from each strain were used for the library construction at the Bioscience Core Lab at KAUST following the manufacturer’s instruction (Pacific Biosciences) [37, 38]. The sequencing was also performed at the Bioscience Core Lab using a PacBio RS II sequencing platform (Pacific Biosciences). The large-insert libraries were sequenced in single-molecule real-time (SMRT) sequencing cells using P6-C4 chemistry.

### De novo Assembly of the Genome Sequencing Data

The row reads of each isolate generated by the PacBio RSII platform were *de novo* assembled and polished with HGAP3/Quiver [39]. The overlapping ends were visually checked by using Gepard v1.40, which would help indicates the circular genomes [40]. Circular closure was performed by using Minimus2 (http://amos.sourceforge.net/wiki/index.php/Minimus2) to trim the ends and permute the genome to begin at the DnaA gene (identified by BLAST), followed by Quiver-based error correction for a final closed genome. We used default parameters for Minimus2 [41].

### Genome Annotation

The FGENESB_annotator was used to predict the presence of likely genes in the genomes of the bacterial isolates (http://www.softberry.com/berry.phtml?topic=fgenesb&group=programs&subgroup=gfindb) [42]. Recently, all known cellulases were classified based on sequence comparison into 16 glycoside-hydrolase (GH) orthologous groups [43]. Since each GH group in the CAZy database is known to have a corresponding Pfam domain (Table S5) [44], the Pfam annotation was used for the identification of cellulase genes in each strain obtained in this study. The Pfam annotation of the deduced amino acid sequences of predicted genes was conducted at the Pfam-A database using the hmmscan program in HMMER (v3.0). The annotations of GH families shown in Table S5 were used to extract candidate cellulase genes, with an E-value cutoff of <1.0e-60 [45].

### Multilocus Sequence Typing (MLST) Analysis

The phylogenetic relationships between the isolates and other *Bacillus* species were determined by multilocus sequence typing (MLST). The amino acid sequences of thirteen housekeeping genes from *Bacillus licheniformis* Table 4 (adk, ccpA, recF, rpoB and sucC) [46], *Bacillus subtilis* WB800N (glpF, ilvD, pta, purH, pycA, rpoD and tpiA) [47] and *Bacillus anthracis CZC5* (gmk) [48] were obtained from PubMLST (http://pubmlst.org/). These thirteen genes are all the gene sets which linked to *Bacillus* in PubMLST, except for apo0A gene which didn’t find in the outgroup genome (*Staphylococcus aureus* subsp. *aureus* NCTC8325).

To perform the MLST analysis, the protein sequences of twenty-two different *Bacillus* species (Table S6) were obtained from the assembly database at the National Center for Biotechnology Information (https://www.ncbi.nlm.nih.gov/) and converted to the database format with the makeblastdb program of the Blast + package version 2.2.31 [49]. For three isolates obtained in this study, amino acid sequences of predicted genes were converted to the BLASTp database format. The identification of housekeeping genes for MLST analysis was conducted via BLASTp searches using thirteen housekeeping gene sequences obtained from PubMLST as a query against the protein databases of each *Bacillus* species. The sequences of the top-scoring hit with an E-value lower than 1.0e-80 and 100 % query coverage were selected from the *Bacillus* genomes for each of thirteen genes. The selected genes were then used as query for the reciprocal Blastp search against proteomes of *B. licheniformis* DSM 13 (GenBank assembly accession: GCF_000011645.1), *B. antthracis* CZC5 (GCF_000534935.1) and *B. subtilis* WB800N (GCF_003610955.1) and confirmed their orthology. The sequence alignment of each gene was conducted using MAFFT version 7.394 with default parameters, and the obtained alignments were concatenated to a single alignment manually [50]. The phylogenetic tree was constructed by the neighbor-joining method with MEGA7 [51]. Branching quality was evaluated by using a bootstrapping confidence value with 1,000 replicates [52].

### RNA Extraction

The isolates were cultured under two different types of culture conditions: cellulase-inducing and non-inducing conditions. Under inducing conditions, the isolates were cultured in media composed of 0.02 % peptone, 0.2 % K_2_HPO_4_, 0.05 % MgSO_4_•7H_2_O, and 0.2 % NaNO_3_ with Whatman No.1 filter paper. In the non-inducing conditions, the filter paper was excluded from the media. Cellulase activities were examined in both conditions with the method described in the Measurement of Cellulase Activity section.

Total RNA was extracted from each of these conditions after 72 h of incubation when it reached the maximum cellulase activity by using a QIAGEN RNeasy mini Kit (Qiagen, Valencia, CA) according to the manufacturer’s protocol. Total RNA quality and concentrations were determined using the Agilent RNA 6000 Pico kit (Agilent, Santa Clara, CA) in a 2100 Bioanalyzer (Agilent). Paired-end libraries with approximate average insert lengths of 200 base pairs were synthesized using the Genomic Sample Prep kit (Illumina, San Diego, CA) according to the manufacturer’s instructions. Libraries were sequenced on the Illumina HiSeq 4000 platform (Illumina, San Diego, CA) with support from the KAUST Bioscience Core laboratory [53].

### Expression Analysis in the Isolates

The nucleotide sequences of ORF region of genes predicted with FGENESB program from the genome sequences were employed to build an index using the bowtie2-build program in the bowtie2 package [54]. Only one side of the paired-end reads generated for each isolate in the RNA-seq experiments described above were aligned to the sequence index by using the Bowtie2 alignment program. The gene expression rate was determined in Reads Per Kilobase of transcript per Million mapped reads (RPKM) units with the following RPKM equation, where the number of short reads mapped onto each ORF region of predicted gene (r_g_) was normalized and divided by the feature length (fl._g_) multiplied by the total number of mapped reads from the sequencing run (R) [55]:

$$\text{R}\text{P}\text{K}\text{M}= \frac{{\text{r}}_{g}\times {10}^{9}}{{fl}_{g}\times \text{R}}$$

### Operon Structure Identification

The FGENESB_annotator web server was used under the default setting to predict the operon structure using the extracted genes as the input. The expression rate of the genes in the operons was determined by the same method described in the expression analysis in the isolates section.

## Supplementary Information

**Additional file 1. **Supplemental Materials. Description of data: Figures S1 to S3; Tables S1 to S6

## Data Availability

PacBio sequencing data and Illumina RNA-seq data have been deposited to the DDBJ Sequence Read Archive under accession numbers DRR228801-DRR228806, and nucleotide sequences of genome assemblies and predicted genes are available in the DDBJ/ENA/GenBank nucleotide database under accession numbers AP023088-AP023090. PB1, SB2 and SB3 are also aliased as RSC (Red Sea Cellulase-producing bacterial strain)-1, RSC-2 and RSC-3 respectively in these databases. CAZy (http://www.cazy.org/), PubMLST (http://pubmlst.org/), Pfam (https://pfam.xfam.org/) and NCBI (https://www.ncbi.nlm.nih.gov/) were used for obtaining reference data in this study, and these databases can be accessed openly.

## Abbreviations

NM: Nutrient Media
CMC: Carboxymethyl Cellulose
ORF: Open Reading Frame
RPM: Revolutions Per Minute
GH: Glycoside hydrolysis
FPU: Filter paper unit
RPKM: Reads Per Kilobase of transcript per Million mapped reads
MLST: Multilocus Sequence Typing
