# Supplementary material for: Genome sequencing and identification of cellulase genes in Bacillus paralicheniformis strains from the Red Sea
Source: BMC Microbiol. 2021 Sep 22;21:254. doi: 10.1186/s12866-021-02316-w (PMC8456639; doi:10.1186/s12866-021-02316-w)
Supplement: Supplementary file 1 — Additional file 1. Supplemental Materials. Description of data: Figures S1 to S3; Tables S1 to S6 [file 12866_2021_2316_MOESM1_ESM.pdf]

Supplemental Material

Genome sequencing and identification of cellulase genes in *Bacillus paralicheniformis* strains from the Red Sea

Siham Fatani, Yoshimoto Saito, Mohammed Alarawi, Takashi Gojobori, Katsuhiko Mineta

Supplemental Tables
Table S1 Whole-genome sequencing of the three bacterial isolates.
Table S2 Comparative expression analysis of genes involved in the operon with cellulase genes in the isolates.
Table S3 Number of RNA-seq reads mapped on ORF regions.
Table S4 Likelihood of β-glucosidases by SignalP.
Table S5 Glycoside hydrolase families including cellulases in CAZy.
Table S6 Reference species for multilocus sequence typing (MLST).

Supplemental Figures
Fig. S1 The degradation of filter paper after 96 hours of incubation at 30 ºC.
Fig. S2 Growth test of the isolates in NM and CMC broth under various salinity conditions. (a) growth in NM broth after 24h culturing, (b) growth in NM broth after 48h culturing, (c) growth in CMC broth after 48h culturing.
Fig. S3 Multiple sequence alignment of GENE_2741 (PB1), GENE_174 (SB2), GENE_1822 (SB3) and GENE_1823 (SB3).

TABLE S1 Whole-genome sequencing of the three bacterial isolates.

**Isolates**	**PB1**	**SB2**	**SB3**
Genome size (bp)	4,318,221	4,318,038	4,317,481
No. of genes	4,362	4,441	4,675
No. of cellulase genes	10	10	11

TABLE S2 Comparative expression analysis of genes involved in the operon with cellulase genes in the isolates.

SB2				
**Operon**	**Gene**	**Inducing (RPKM)**	**Non-inducing (RPKM)**	**Ratio**
Operon-1	GENE_2609	188.18	81.24	2.31
	GENE_2610	75.03	110.57	0.67
Operon-2	GENE_2582	66.47	37.67	1.76
	GENE_2583	36.99	36.69	1.00
Operon-3	GENE_3143	11.81	107.74	0.10
	GENE_3144	0.83	5.09	0.16
	GENE_3145	15.46	7.48	2.06
Operon-4	GENE_2557	7.90	2.81	2.80
	GENE_2558	6.038	4.032	1.49
Operon-5	GENE_958	949.02	2.28	414.79
	GENE_959	525.08	1.49	351.57
	GENE_960	209.62	1.73	120.66
	GENE_961	365.83	5.24	69.78
Operon-6	GENE_2941	46.05	0.75	61.19
	GENE_2942	30.19	0.77	38.78
	GENE_2943	32.20	0.68	46.97
	GENE_2944	21.60	1.88	11.48
Operon-7	GENE_443	48.47	261.73	0.18
	GENE_444	3.74	4.48	0.83
	GENE_445	10.24	1.59	6.41
Operon-8	GENE_173	1.23	0.35	3.47
	GENE_174	4.51	0.65	6.87

SB3				
**Operon**	**Gene**	**Inducing (RPKM)**	**Non-inducing (RPKM)**	**Ratio**
Operon-1	GENE_4396	3.22	0.17	18.22
	GENE_4397	24.84	1.51	16.43
Operon-2	GENE_4368	3.84	0.08	45.97
	GENE_4369	347.43	17.03	20.40
Operon-3	GENE_4370	679.75	27.91	24.34
	GENE_286	99.81	1.94	51.27
	GENE_287	618.26	38.89	15.89
Operon-4	GENE_288	157.70	7.61	20.70
	GENE_4343	19.90	0	inducing specific
Operon-5	GENE_4344	43.50	0.22	191.50
	GENE_2665	21.05	6.56	3.20
	GENE_2666	2.11	0.07	27.89
	GENE_2667	0.94	0	inducing specific
	GENE_2668	2.12	0.32	6.58
Operon-6	GENE_73	164.23	0.56	288.68
	GENE_74	261.19	0.90	288.45
	GENE_75	308.11	0.61	501.54
	GENE_76	348.87	0.07	4481.06
	GENE_77	460.98	0.49	934.59
	GENE_78	583.39	0.75	770.35
	GENE_79	551.44	0.68	802.63
Operon-7	GENE_2102	4.22	2.26	1.86
	GENE_2103	53.80	0.77	69.43
	GENE_2104	54.92	0.54	101.30
Operon-8	GENE_1821	43.32	2.07	20.86
	GENE_1822	16.63	4.60	3.61
	GENE_1823	17.40	3.62	4.79

The orders of genes inside the operons were identical to those in Fig 4.

Abbreviations: **inducing**, expression (RPKM) in the cellulase inducing condition; **non-inducing**, expression in the non-inducing condition; **ratio**, ratio of expression in the inducing condition / expression in the non-inducing condition.

TABLE S3 Number of RNA-seq reads mapped on ORF regions.

	PB1		SB2		SB3	
	*inducing	*non-inducing	inducing	non-inducing	inducing	non-inducing
No. of reads	9,512	6,956,050	4,883,276	4,909,236	69,604,052	30,615,468

* **inducing**, under the cellulase-inducing condition; **non-inducing**, under the condition in which no cellulases are induced. See materials and Methods.

TABLE S4 Likelihood of β-glucosidases by SignalP.

Isolates			Protein type	Signal peptide (Sec/SPI)	TAT signal peptide (Tat/SPI)	Lipoprotein signal peptide (Sec/SPII)	Other
**PB1**	**SB2**	**SB3**					
GENE_769	GENE_2609	GENE_4396	GH1	0.0951	0.0157	0.0177	0.8715
GENE_743	GENE_2582	GENE_4369	GH1	0.0547	0.0045	0.0124	0.9284
GENE_1298	GENE_3145	GENE_288	GH1	0.0642	0.0185	0.0193	0.898
GENE_718	GENE_2557	GENE_4343	GH1	0.3373	0.2209	0.047	0.3947
GENE_3516	GENE_958	GENE_2665	GH1	0.2345	0.1663	0.0119	0.5873
GENE_1100	GENE_2942	GENE_74	GH3	*0.9704	0.0042	0.0243	0.0011

*Cleavage site between pos. 28 and 29: AEA-KQ. Probability: 0.6689

TABLE S5 Glycoside hydrolase families including cellulases in CAZy.

GH family	Enzyme name (Activity)
GH1 PF00232	β-glucosidase; β-galactosidase; β-mannosidase; β-glucuronidase; β-xylosidase; β-D-fucosidase; phlorizin hydrolase; exo-β-1,4-glucanase; 6-phospho-β-galactosidase; 6-phospho-β-glucosidase; strictosidine β-glucosidase; lactase; amygdalin β-glucosidase; prunasin β-glucosidase; vicianin hydrolase; raucaffricine β-glucosidase; thioglucosidase; β-primeverosidase; isoflavonoid 7-O-β-apiosyl-β-glucosidase; ABA-specific β-glucosidase; DIMBOA β-glucosidase; β-glycosidase; hydroxyisourate hydrolase.
GH3 PF00933	β-glucosidase; xylan 1,4-β-xylosidase; β-glucosylceramidase; β-N-acetylhexosaminidase; α-L-arabinofuranosidase; glucan 1,3-β-glucosidase; glucan 1,4-β-glucosidase; isoprimeverose-producing oligoxyloglucan hydrolase; coniferin β-glucosidase; exo-1,3-1,4-glucanase; β-N-acetylglucosaminide phosphorylases.
GH5 PF00150	Endo-β-1,4-glucanase / cellulase; endo-β-1,4-xylanase; β-glucosidase; β-mannosidase; β-glucosylceramidase; glucan β-1,3-glucosidase; licheninase; exo-β-1,4-glucanase / cellodextrinase; glucan endo-1,6-β-glucosidase; mannan endo-β-1,4-mannosidase; cellulose β-1,4-cellobiosidase; steryl β-glucosidase; endoglycoceramidase; chitosanase; β-primeverosidase; xyloglucan-specific endo-β-1,4-glucanase; endo-β-1,6-galactanase; hesperidin 6-O-α-L-rhamnosyl-β-glucosidase; β-1,3-mannanase; arabinoxylan-specific endo-β-1,4-xylanase; mannan transglycosylase.
GH6 PF01341	Endoglucanase; cellobiohydrolase
GH7 PF00840	Endo-β-1,4-glucanase; reducing end-acting cellobiohydrolase; chitosanase; endo-β-1,3-1,4-glucanase
GH8 PF01270	Chitosanase; cellulase; licheninase; endo-1,4-β-xylanase; reducing-end-xylose releasing exo-oligoxylanase
GH9 PF00759	Endoglucanase; endo-β-1,3(4)-glucanase / lichenase-laminarinase; β-glucosidase; lichenase / endo-β-1,3-1,4-glucanase; exo-β-1,4-glucanase / cellodextrinase; cellobiohydrolase; xyloglucan-specific endo-β-1,4-glucanase / endo-xyloglucanase; exo-β-glucosaminidase
GH12 PF01670	Endoglucanase; xyloglucan hydrolase; β-1,3-1,4-glucanase; xyloglucan endotransglycosylase
GH26 PF02156	β-mannanase; exo-β-1,4-mannobiohydrolase; β-1,3-xylanase; lichenase / endo-β-1,3-1,4-glucanase; mannobiose-producing exo-β-mannanase
GH30 PF02055	Endo-β-1,4-xylanase; β-glucosidase; β-glucuronidase; β-xylosidase; β-fucosidase; glucosylceramidase; β-1,6-glucanase; glucuronoarabinoxylan endo-β-1,4-xylanase; endo-β-1,6-galactanase; [reducing end] β-xylosidase
GH44 PF12891	Endoglucanase; xyloglucanase
GH45 PF02015	Endoglucanase
GH48 PF02011	Reducing end-acting cellobiohydrolase; endo-β-1,4-glucanase; chitinase
GH61 PF03443	copper-dependent lytic polysaccharide monooxygenases (LPMOs) that have oxidative cleavage of cellulose.

TABLE S6 Reference species for multilocus sequence typing (MLST).

Reference species	Accession number
Bacillus paralicheniformis Bac48	ASM299394v1
Bacillus paralicheniformis Bac84	ASM299392v1
Bacillus paralicheniformis ATCC 9945a	ASM40888v1
Bacillus paralicheniformis KJ-16	ASM104248v2
Bacillus paralicheniformis 167/2	ASM193954v1
Bacillus licheniformis DSM 13	ASM1164v1
Bacillus sonorensis SRCM101395	ASM220201v1
Bacillus atrophaeus SRCM101359	ASM217349v1
Bacillus subtilis subsp. *inaquosorum* DE111	ASM153478v1
Bacillus siamensis SCSIO 05746	ASM285053v1
Bacillus vallismortis	ASM211380v1
Bacillus velezensis FZB42	ASN1578v1
Bacillus altitudinis B388	ASM78942v2
Bacillus litoralis Bac94	ASM366782v1
Bacillus luciferensis CH01	ASM171275v1
Bacillus sp. FDAARGOS_527	ASM381212v1
Bacillus anthracis Ames	AMS784v1
Bacillus thermoamylovorans	ASM309621v1
Bacillus macauensis ZFHKF-1	ASM26986v1
Bacillus clausii KSM-K16	ASM982v1
Bacillus patagoniensis	ASM201970v1
Staphylococcus aureus NCTC_8325	ASM1342v1

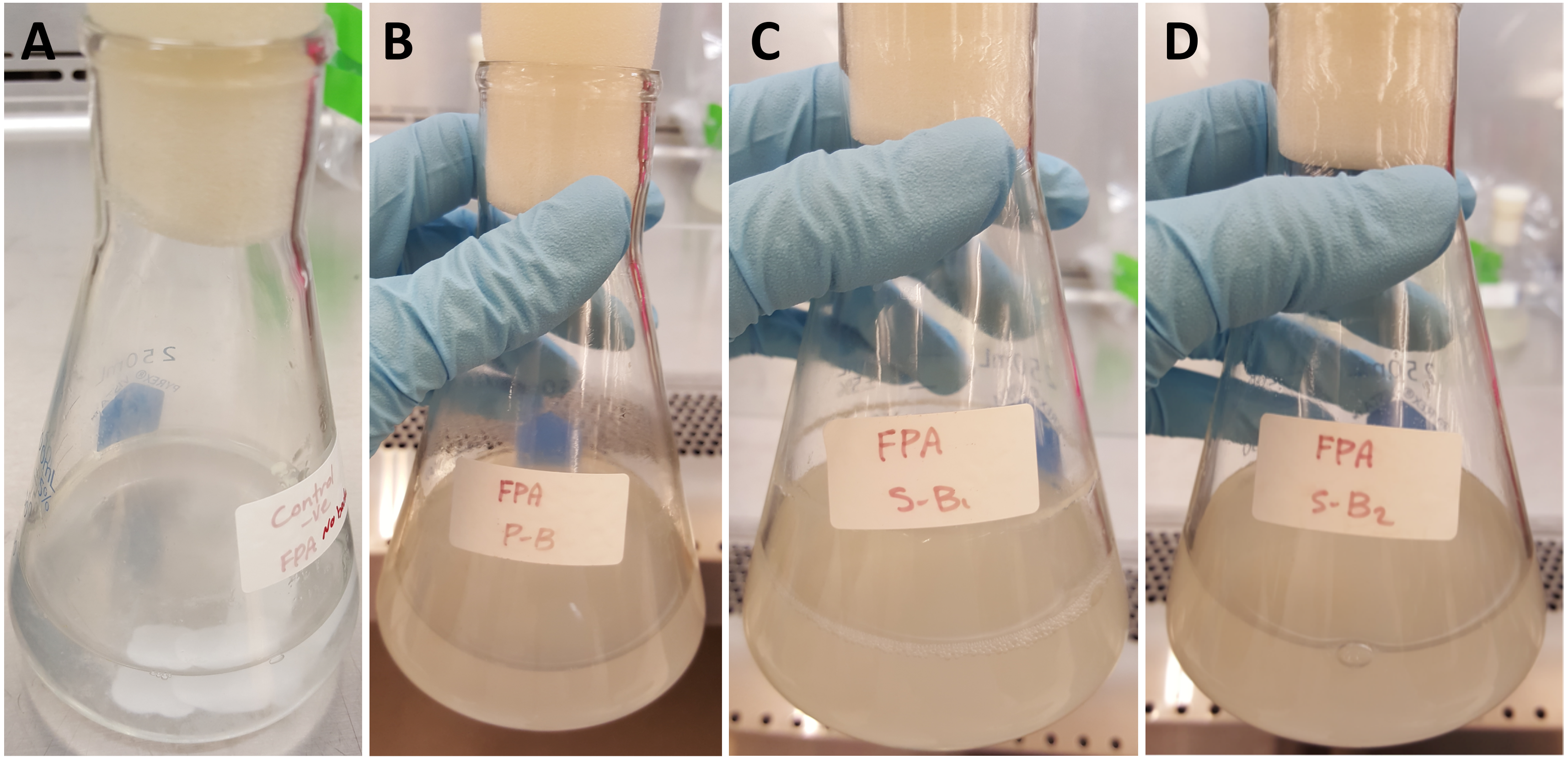

FIG S1 The degradation of filter paper after 96 hours of incubation at 30°C. (A) Negative control, (B) PB1, (C) SB2 and (D) SB3.

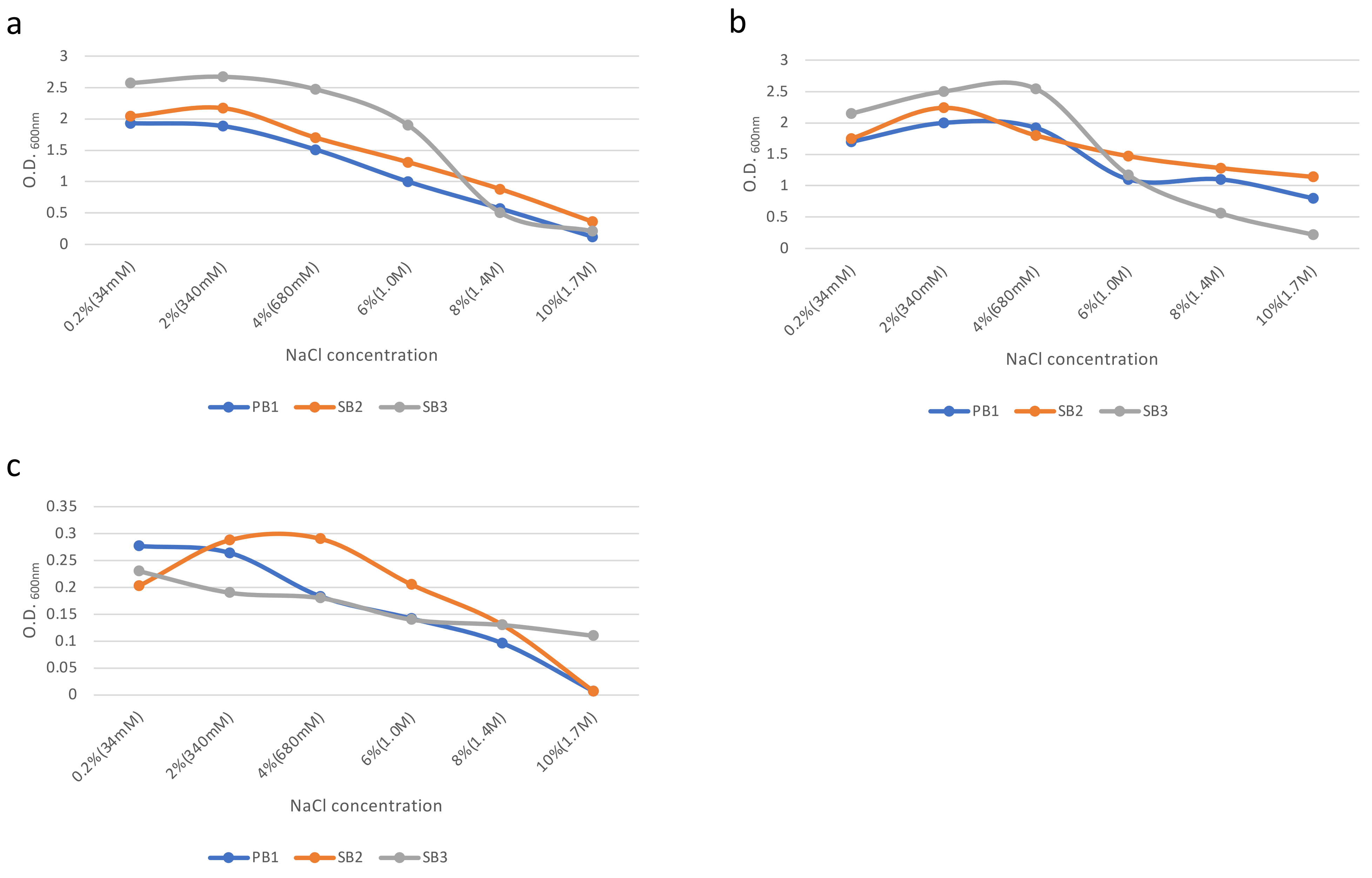

FIG. S2 Growth test of the isolates in NM and CMC broth under various salinity conditions. (a) growth in NM broth after 24h culturing, (b) growth in NM broth after 48h culturing, and (c) growth in CMC broth after 48h culturing.

```
PB1.GENE_2741   MYNKTRFMQLYEQIKNPQNGYFSPEGIPYHSVETLICEAPDYGHMTTSEAYSYWLWLEAM
SB2.GENE_174    MYNKTRFMQLYEQIKNPQNGYFSPEGIPYHSVETLICEAPDYGHMTTSEAYSYWLWLEAM
SB3.GENE_1822   MYNKTRFMQLYEQIKNPQNGYFSPEGIPYHSVETLICEAPDYGHMTTSEAYSYWLWLEAM
SB3.GENE_1823   ------------------------------------------------------------


PB1.GENE_2741   YGRYTQDWSKLEAAWDNMEKYIIPVNEGDGNEEQPTMNYYNPSSPATYAAEHRYPDLYPS
SB2.GENE_174    YGRYTQDWSKLEAAWDNMEKYIIPVNEGDGNEEQPTMNYYNPSSPATYAAEHRYPDLYPS
SB3.GENE_1822   YGRYTQDWSKLEAAWDNMEKYIIPVNEGDGNEEQPTMNYYNPSSPATYAAEHRYPDLYPS
SB3.GENE_1823   ------------------------------------------------------------


PB1.GENE_2741   ALTGQYPAGNDPLDSELRSTYGSNETYLMHWLLDVDNWYGFGNLLNPSHTAVYVNTYQRG
SB2.GENE_174    ALTGQYPAGNDPLDSELRSTYGSNETYLMHWLLDVDNWYGFGNLLNPSHTAVYVNTYQRG
SB3.GENE_1822   ALTGQYPAGNDPLDSELRSTYGSNETYLMHWLLDVDNWYGFGNLLNPSHTAVYVNTYQRG
SB3.GENE_1823   ------------------------------------------------------------


PB1.GENE_2741   EQESVWETVPHPSQDNQTFGKPNEGFMSLFTKENQAPAPQWRYTNATDADARAVQAMYWA
SB2.GENE_174    EQESVWETVPHPSQDNQTFGKPNEGFMSLFTKENQAPAPQWRYTNATDADARAVQAMYWA
SB3.GENE_1822   EQESVWETVPHPSQDNQTFGKPNEGFMSLFTKENQAPAPQWRYTNATDADARAVQAMYWA
SB3.GENE_1823   ------------------------------------------------------------


PB1.GENE_2741   MQWGYSNTKYLEKAKKMGDFLRYGMYDKYFQEIGSAADGSPSRGTGKNACHYLMAWYTAW
SB2.GENE_174    MQWGYSNTKYLEKAKKMGDFLRYGMYDKYFQEIGSAADGSPSRGTGKNACHYLMAWYTAW
SB3.GENE_1822   MQWGYSNTKYLEKAKKMGTFSVTACMTNTFKRLEALL-----------------------
SB3.GENE_1823   ------------------------MYDKYFQEIGSAADGSPSRGTGKNACHYLMAWYTAW
                                           : *:.: :

PB1.GENE_2741   GGGLGQYANWAWRIGASHVHQGYQNPVASYALSTAEGGLVPNSSTARSDWEQALKRQLEL
SB2.GENE_174    GGGLGQYANWAWRIGASHVHQGYQNPVASYALSTAEGGLVPNSSTARSDWEQALKRQLEL
SB3.GENE_1822   --------------------------------------------TAR-------------
SB3.GENE_1823   GGGLGQYANWAWRIGASHVHQGYQNPVASYALSTAEGGLVPNSSTARSDWEQALKRQLEL
                                                            ***

PB1.GENE_2741   YTWLLSSEGAVAGGATNSWNGSYSAYPQNVSTFYGMAYTEAPVYHDPPSNNWFGMQVWPL
SB2.GENE_174    YTWLLSSEGAVAGGATNSWNGSYSAYPQNVSTFYGMAYTEAPVYHDPPSNNWFGMQVWPL
SB3.GENE_1822   ------------------------------------------------------------
SB3.GENE_1823   YTWLLSSEGAVAGGATNSWNGSYSAYPQNVSTFYGMAYTEAPVYHDPPSNNWFGMQVWPL


PB1.GENE_2741   ERVAELYYIFAEKGDKSSENFQMAKHVIEKWIAYSLDYVFVGERPVTDEEGYYLNEAGER
SB2.GENE_174    ERVAELYYIFAEKGDKSSENFQMAKHVIEKWIAYSLDYVFVGERPVTDEEGYYLNEAGER
SB3.GENE_1822   ------------------------------------------------------------
SB3.GENE_1823   ERVAELYYIFAEKGDKSSENFQMAKHVIEKWIAYSLDYVFVGERPVTDEEGYYLNEAGER


PB1.GENE_2741   VLGGQNPQIAVQSDPGEFWIPANLEWSGQPDPWKGFDSFTGNPGLHVTTKNPSQDVGVLG
SB2.GENE_174    VLGGQNPQIAVQSDPGEFWIPANLEWSGQPDPWKGFDSFTGNPGLHVTTKNPSQDVGVLG
SB3.GENE_1822   -------------------LPAELE-----------------------------------
SB3.GENE_1823   VLGGQNPQIAVQSDPGEFWIPANLEWSGQPDPWKGFDSFTGNPGLHVTTKNPSQDVGVLG
                                   :**:**

PB1.GENE_2741   SYIKTLVFFAAGTKAETGGFTALGNKAKNVAKELLDAAWNKNDGIGIAAEEEHEDYIRYF
SB2.GENE_174    SYIKTLVFFAAGTKAETGGFTALGNKAKNVAKELLDAAWNKNDGIGIAAEEEHEDYIRYF
SB3.GENE_1822   ------------------------------------------------------------
SB3.GENE_1823   SYIKTLVFFAAGTKAETGGFTALGNKAKNVAKELLDAAWNKNDGIGIAAEEEHEDYIRYF


PB1.GENE_2741   TKEVYFPNGWSGKNGQGNTIPGSNTVPSDPAKGGNGVYISHADLRPKIKNDPMWPYLENK
SB2.GENE_174    TKEVYFPNGWSGKNGQGNTIPGSNTVPSDPAKGGNGVYISHADLRPKIKNDPMWPYLENK
SB3.GENE_1822   ------------------------------------------------------------
SB3.GENE_1823   TKEVYFPNGWSGKNGQGNTIPGSNTVPSDPAKGGNGVYISHADLRPKIKNDPMWPYLENK


PB1.GENE_2741   YQTSWNPNTGKWENGLPTFVYHRFWSQVDMATAYAEYDRLIGNA
SB2.GENE_174    YQTSWNPNTGKWENGLPTFVYHRFWSQVDMATAYAEYDRLIGNA
SB3.GENE_1822   --------------KMPVII------------------------
SB3.GENE_1823   YQTSWNPNTGKWENGLPTFVYHRFWSQVDMATAYAEYDRLIGNA
```

FIG S3 Multiple sequence alignment of GENE_2741 (PB1), GENE_174 (SB2), GENE_1822 (SB3) and GENE_1823 (SB3).
